# Brain dynamic audio stimulation for subjective sleep disturbances in healthcare professionals: a single-arm pilot feasibility study

**DOI:** 10.3389/fnins.2026.1893783

**Published:** 2026-08-21

**Authors:** Hong-Min Lin, Ming-Chung Ho, Yan-Lin Zhong, Chih-Hung Tsai, Wan-Ting Hsieh

**Affiliations:** 1 Department of Family Medicine, Chi-Mei Medical Center, Tainan, Taiwan; 2 Department of Physics, National Kaohsiung Normal University, Kaohsiung, Taiwan

**Keywords:** auditory neuromodulation, brain dynamic audio stimulation, electroencephalography, healthcare professionals (HCP), sleep disturbances, sleep initiation

## Abstract

**Background:**

Insomnia and sleep disturbances are highly prevalent among healthcare professionals and pose risks to patient safety. Brain Dynamic Audio Stimulation (BDAS) is a non-invasive, open-loop auditory intervention intended to facilitate sleep initiation. This single-arm pilot feasibility study examined the feasibility and preliminary signal of a 14-day BDAS intervention in healthcare professionals with subjective sleep disturbances.

**Methods:**

Of 30 healthcare professionals assessed for eligibility, 15 were enrolled. Participants received one 30-min BDAS session nightly for 14 consecutive days. Insomnia severity was assessed using the Insomnia Severity Index (ISI) at baseline and Day 14. Short-duration EEG recordings (30 min, time-locked to BDAS, focused on sleep initiation) were obtained at both time points. Pre–post changes were analyzed using paired-sample tests; EEG outcomes were considered exploratory.

**Results:**

All 15 participants completed the intervention (100% retention; no adverse events). Mean ISI scores decreased from 10.00 (SD 5.55) to 4.27 (SD 2.69) (mean change −5.73; 95% CI − 8.15 to −3.32; Cohen’s *d*z = 1.31; *p* < 0.001). Exploratory EEG analyses showed reductions in sleep onset latency (828 s to 480 s; *d*z = 0.89; *p* = 0.004) and increases in 30-min sleep efficiency (34.8 to 46.8%; *d*z = 0.57; *p* = 0.045). All predefined feasibility progression criteria were met.

**Conclusion:**

BDAS demonstrated feasibility and acceptability in a healthcare professional setting. The observed within-participant improvements in insomnia severity and exploratory EEG parameters provide a preliminary signal and effect size estimates to inform future randomized controlled trials. Because the design was single-arm and uncontrolled, these within-participant changes cannot be attributed to BDAS specifically.

## 1 Introduction

Insomnia and sleep disturbances are highly prevalent among healthcare professionals, who are frequently exposed to shift work, irregular schedules, high workload, and sustained psychosocial stress. Epidemiological studies indicate that approximately one-third to nearly half of healthcare workers experience clinically significant insomnia symptoms, particularly among nurses and frontline staff (Liu et al., 2025; Pappa et al., 2020). Sleep problems in healthcare professionals have been associated with impaired cognitive and emotional functioning and may adversely affect occupational performance and patient safety (Morin and Benca, 2012). Although pharmacological treatments may provide short-term relief, their use is limited by adverse effects, tolerance, and dependence (Kripke, 2018). Higher perceived stress has been linked to greater insomnia severity and increased hypnotic medication use (De Las Cuevas and Segovia Díaz, 2025), underscoring the need for low-risk, non-pharmacological interventions that address stress-related hyperarousal in occupational settings.

Auditory neuromodulation approaches, including music-based interventions, auditory beat stimulation, and brainwave entrainment, have been explored as potential tools to facilitate sleep initiation and improve sleep quality through modulation of cortical and autonomic activity (Askarpour et al., 2024; Capezuti et al., 2022; Ingendoh et al., 2023; Jespersen et al., 2022). Systematic reviews of binaural beats and related paradigms report heterogeneous and often inconsistent effects on EEG activity and insomnia symptoms, and most studies have been conducted in healthy or experimental populations, limiting clinical generalizability (Ingendoh et al., 2023; Jespersen et al., 2022). More recently, acoustic stimulation protocols such as music therapy, noise-based interventions, and closed-loop auditory stimulation of slow-wave sleep have shown that non-pharmacological sound-based approaches can improve subjective sleep quality and insomnia severity in some clinical and subclinical populations, although effects on sleep efficiency and total sleep time remain mixed (Capezuti et al., 2022; Chang et al., 2012; Dudysová et al., 2024; Tegeler et al., 2020; Wang et al., 2025). Overall, the emerging evidence suggests that acoustic stimulation is a promising adjunctive strategy for insomnia and sleep disturbances, but optimal stimulation parameters, mechanisms, and target populations remain to be clarified.

More recently, a randomized controlled trial in healthcare workers evaluated a closed-loop, real-time EEG-guided acoustic neuromodulation approach and reported improvement in perceived stress, with insomnia and anxiety included as secondary outcomes (Tegeler et al., 2025). This closed-loop architecture differs technically from open-loop BDAS. Brain Dynamic Audio Stimulation (BDAS) is an open-loop auditory intervention whose proprietary stimulus-generation framework was developed under an experimental brain-dynamics hypothesis and was informed by nonlinear analytical methods. This framework describes the design rationale and does not constitute evidence of a demonstrated neurophysiological mechanism. Preliminary EEG-based work in healthy adults has reported sleep-stage transitions during BDAS exposure; however, these findings are exploratory, derive from small non-clinical samples, and do not establish cortical entrainment or a specific mechanism of action (Ho et al., 2025). Given its non-invasive and non-pharmacological nature, BDAS supports further evaluation in healthcare professionals reporting subjective sleep disturbances.

The present study therefore examines BDAS as a non-pharmacological approach for healthcare professionals with subjective sleep disturbances, focusing on insomnia symptoms and early sleep-related EEG dynamics. In light of limited and heterogeneous evidence on acoustic stimulation in clinical populations (Capezuti et al., 2022; Jespersen et al., 2022; Wang et al., 2025), this single-group pretest–posttest pilot feasibility study evaluates whether a two-week BDAS intervention is associated with changes in perceived insomnia severity and short-duration EEG-derived sleep-related parameters, particularly sleep initiation and short-term sleep efficiency. By assessing subjective and objective sleep outcomes before and after a structured intervention, this exploratory investigation aims to generate preliminary feasibility data and effect size estimates to inform the design of future randomized controlled trials, rather than to establish definitive efficacy or mechanisms of action.

## 2 Methods

### 2.1 Study design and ethical approval

This study was designed as a prospective pilot feasibility study using a single-group pretest–posttest design to examine the effects of BDAS on insomnia severity and sleep-related parameters in healthcare professionals. The study was conducted at Chi Mei Medical Center and was registered on ClinicalTrials.gov (PRS Protocol Registration Preview; Identifier: NCT07252128). The study protocol was approved by the Institutional Review Board of Chi Mei Medical Center (approval number: 11404–012) on 14 August, 2025. All procedures were conducted in accordance with the Declaration of Helsinki, and written informed consent was obtained from all participants prior to enrollment. No changes were made to the study protocol, eligibility criteria, or outcome assessments after trial commencement. Given the pilot feasibility design and small sample size, all analyses were considered exploratory and primarily aimed at estimating effect sizes and assessing feasibility rather than establishing definitive efficacy.

### 2.2 Participants

Healthcare professionals aged 20–65 years were recruited from Chi Mei Medical Center. Eligibility criteria required participants to report subjective sleep disturbance or poor sleep quality persisting for at least 1 month and to be willing and able to complete the intervention and study assessments. Exclusion criteria included current use of sedative-hypnotic or psychiatric medications, previously diagnosed sleep disorders such as obstructive sleep apnea or narcolepsy, a history of major psychiatric, neurological, or severe chronic medical illness, significant hearing impairment that could interfere with auditory stimulation, and pregnancy or breastfeeding. Participants were not required to meet formal diagnostic criteria for chronic insomnia; instead, recruitment focused on healthcare professionals with subjectively reported sleep disturbances in routine clinical practice. Eligible individuals received detailed verbal and written information about the study and provided written informed consent before enrollment.

### 2.3 Sample size

As an exploratory pilot study, the target sample size of 15 participants was determined based on feasibility considerations rather than formal power calculations. Although a sample size of 12 participants per group has been proposed as a pragmatic rule of thumb for some pilot trials, sample size should reflect the feasibility objectives and desired estimation precision (Julious, 2005; Sim and Lewis, 2012). For this single-arm study, 15 participants were selected to assess recruitment, retention, intervention acceptability, and measurement feasibility.

### 2.4 Intervention

All participants received the same BDAS intervention in this single-arm study. BDAS is an open-loop auditory stimulation system, and its auditory output is not adjusted in real time using EEG signals. Each participant completed one 30-min BDAS session per day for 14 consecutive days. Participants were instructed to initiate each session within 15 min of going to bed and preparing to sleep, were permitted to fall asleep during stimulation, and were asked to maintain a consistent bedtime schedule across the two-week intervention period.

**Figure 1:**
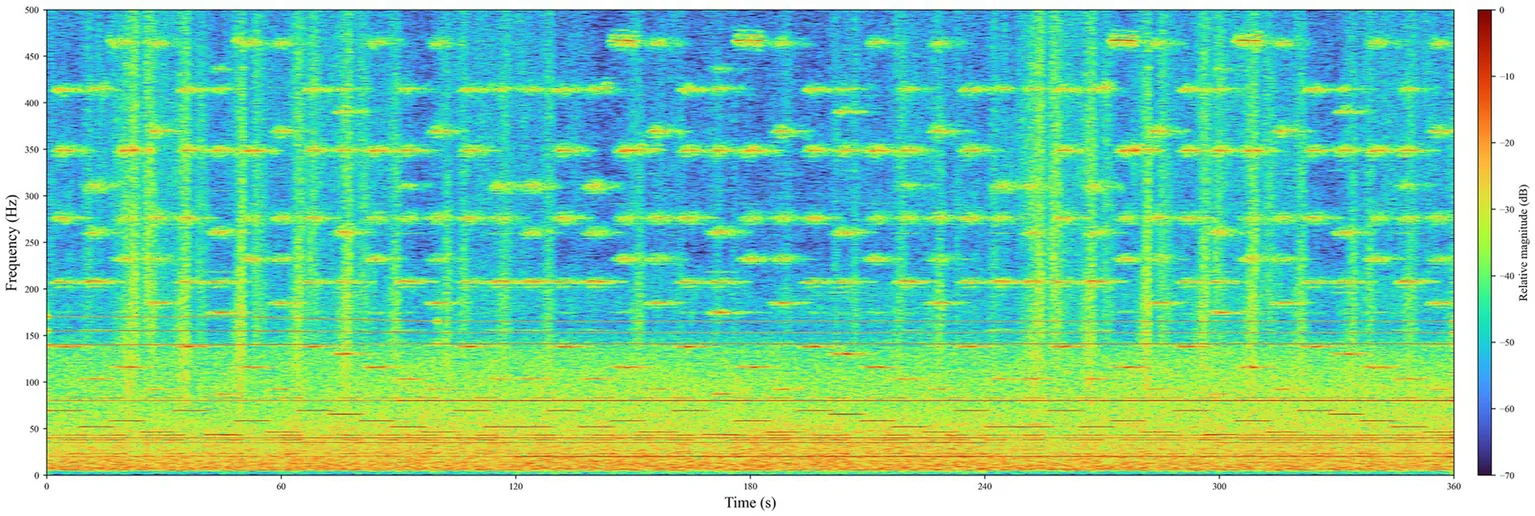
Representative 360-s time-frequency characteristics of the Brain Dynamic Audio Stimulation (BDAS) stimulus. The spectrogram illustrates the temporal evolution of the principal acoustic components throughout the stimulation period. The stimulus combines continuous natural environmental sounds, periodic acoustic components, and low-frequency background sounds (brown noise), demonstrating the representative time-frequency characteristics of the BDAS stimulus.

The same auditory signal was presented simultaneously to both ears through stereo headphones. The BDAS stimulus consisted of continuous natural environmental sounds, including ocean-wave sounds, combined with periodic acoustic components of several predefined frequencies. Low-frequency background sounds (brown noise) were incorporated to produce a continuously varying auditory environment. Frequency and amplitude were continuously modulated throughout the stimulation period according to predefined modulation principles implemented within the BDAS framework. A representative 360-s time-frequency spectrogram of the BDAS stimulus is shown in Figure 1. The intervention was delivered through over-ear headphones using the commercially available NeurVI system (NeurVI, Taiwan). The system used in this study was purchased by Chi Mei Medical Center using institutional funds. Only the playback volume was individualized by allowing each participant to select a comfortable listening level; the stimulation algorithm itself was not individualized. Daily sessions were completed at home, whereas EEG recordings were conducted separately at the hospital at baseline and Day 14. No other structured sleep intervention or hypnotic medication was provided during the study period.

### 2.5 Outcome measures

Subjective insomnia severity was assessed using the Insomnia Severity Index (ISI), a validated seven-item self-report questionnaire with total scores ranging from 0 to 28, with higher scores indicating greater insomnia severity. The ISI was administered at baseline (Day 0, pre-intervention) and after completion of the 14-day intervention period (Day 14, post-intervention). The primary outcome measure was the within-participant change in total ISI score from baseline to post-intervention. For exploratory analyses, participants were stratified according to baseline ISI severity (0–7, no clinically significant insomnia; 8–14, mild insomnia; 15–28, moderate-to-severe insomnia), and pre-post changes within each subgroup were examined descriptively.

Objective sleep-related parameters were assessed using short-duration physiological recordings time-locked to BDAS, following an approach adapted from prior exploratory work (Ho et al., 2025). Sleep onset latency was selected to quantify the interval from audio onset to the first AASM-defined sleep epoch, whereas 30-min sleep efficiency quantified the proportion of the fixed stimulation window spent in any AASM-defined sleep stage. Together, these outcomes characterize sleep initiation and short-duration sleep maintenance within a potentially feasible brief-rest opportunity for healthcare personnel; they were not intended to replace full-night polysomnography.

Physiological data were acquired using a portable 40-channel digital recording system (NuAmps 40-channel DC Amplifier, Compumedics Neuroscan, United States), comprising 32 scalp EEG channels, four EOG channels, two ECG channels, and two submental EMG channels. Scalp electrodes were positioned according to the International 10–10 system. A1 and A2 mastoid electrodes were placed separately from the dedicated reference electrode and ground connection. Electrode impedance was maintained below 5 kOhm, and signals were sampled at 2048 Hz and band-pass filtered between 0.5 and 35 Hz. Recordings were conducted in a quiet, controlled hospital environment. Each session lasted 30 min, began at audio onset, and continued throughout the fixed 30-min stimulation period. This configuration supported AASM-based sleep staging, physiological monitoring, artifact identification, and data-quality assessment.

Sleep staging was performed in 30-s epochs according to American Academy of Sleep Medicine criteria, classifying epochs as wakefulness, N1, N2, N3, or REM sleep when present (Troester et al., 2023). Software-assisted analysis was followed by manual review by trained scorers blinded to the assessment time point. Independent component analysis was used to identify and remove artifactual components. Epochs affected by residual artifacts or signal interruption were excluded from sleep staging. Sleep continuing after the 30-min stimulation period ended was not included in the outcomes.

Spectral decomposition of the standard delta, theta, alpha, and beta bands was considered as an exploratory aim but was not undertaken in the present feasibility report. The EEG outcomes reported here were therefore restricted to sleep-staging-derived indices, namely sleep onset latency and 30-min sleep efficiency. Direct evaluation of cortical entrainment will require quantitative stimulus-synchronized spectral and phase-locking analyses in future controlled studies.

### 2.6 Progression criteria for a future definitive trial

Progression to a future randomized controlled trial will be considered feasible if predefined feasibility and preliminary efficacy thresholds are achieved. Feasibility will be indicated by recruitment of at least 12 participants within the planned recruitment period, retention of ≥80% for intervention completion and outcome assessments, absence of serious BDAS-related adverse events, and early discontinuation due to intervention-related discomfort in ≤20% of participants. Measurement feasibility will be defined as successful acquisition of analyzable EEG data in ≥80% of recording sessions. A preliminary efficacy signal will be considered present if a moderate reduction in ISI scores (Cohen’s d ≥0.5) or statistically significant improvement in at least one sleep-related outcome is observed. These criteria were defined *a priori* to inform planning of a future definitive trial.

### 2.7 Statistical analysis

Statistical analyses were performed using parametric methods. Pre–post changes in ISI total scores were analyzed using paired-sample *t* tests because the same participants were assessed at baseline and after the 14-day intervention. Effect size was calculated as Cohen’s dz., defined as the mean within-participant reduction divided by the standard deviation of the within-participant reduction. Hedges’ gz was additionally reported using a small-sample correction. Given the limited sample size, Wilcoxon signed-rank tests were performed as non-parametric sensitivity analyses. Exploratory subgroup analyses were conducted according to baseline ISI severity and were interpreted descriptively.

Short-duration EEG-derived outcomes were analyzed separately from ISI. Sleep onset latency was defined as the interval from audio onset to the first 30-s epoch scored as any AASM-defined sleep stage. Thirty-minute sleep efficiency was defined as the cumulative duration of all epochs scored as N1, N2, N3, or REM sleep during the active 30-min stimulation window, divided by the fixed 30-min stimulation duration and multiplied by 100. Sleep continuing after stimulation ended was not included. Epochs excluded because of residual artifact or signal interruption were not staged as sleep, but the denominator remained the prespecified 30-min duration. Paired-sample t tests assessed baseline-to-Day 14 changes, with Wilcoxon signed-rank tests as non-parametric sensitivity analyses. These outcomes were exploratory and are not equivalent to conventional whole-night sleep efficiency or a complete assessment of sleep architecture. All tests were two-sided, and *p* < 0.05 was considered statistically significant.

## 3 Results

### 3.1 Participant flow and recruitment

**Figure 2:**
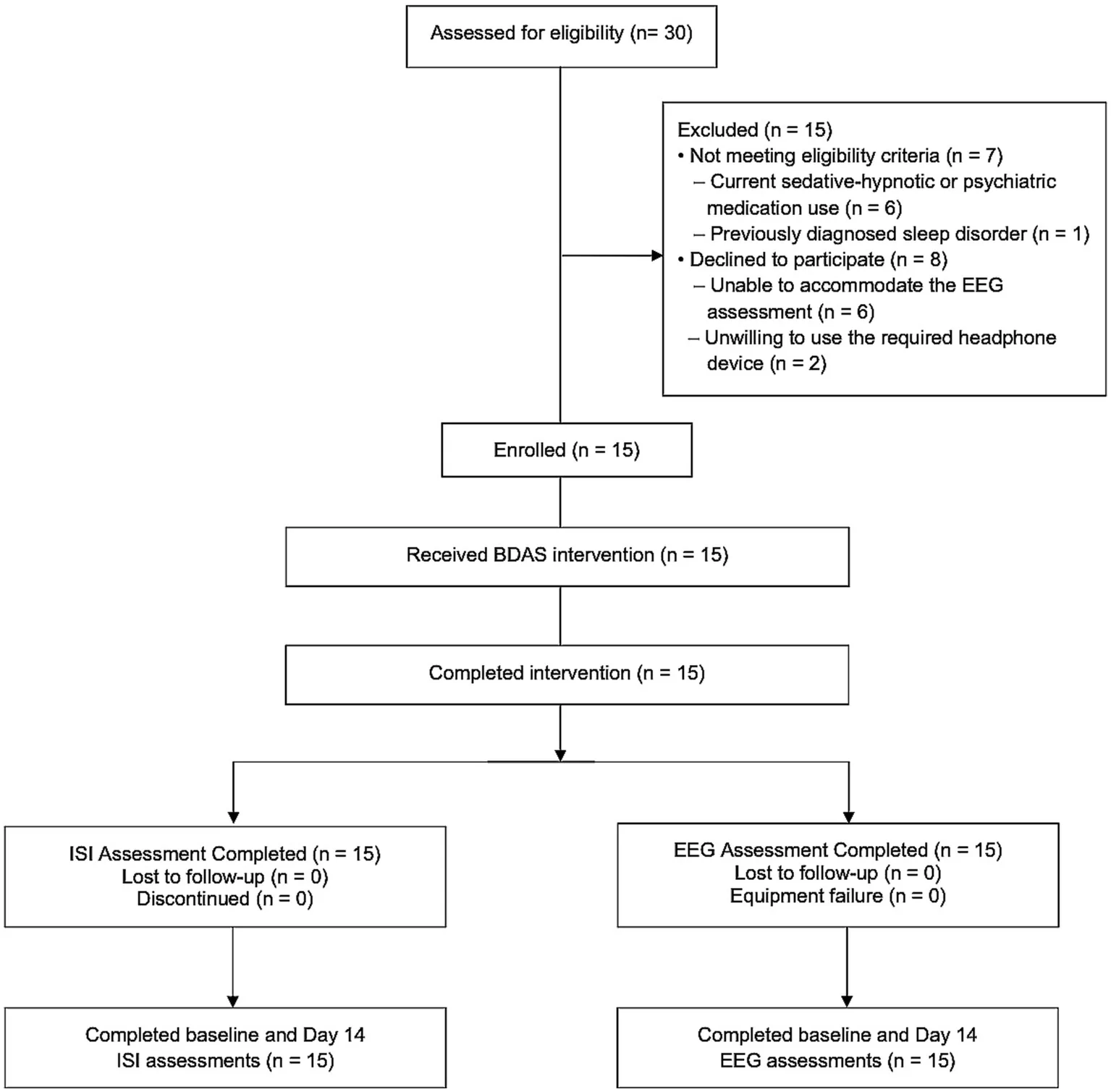
Participant flow diagram of recruitment, intervention completion, and analysis.

Figure 2 presents the flow of participants through the study. A total of 30 healthcare professionals were assessed for eligibility between August 2025 and March 2026. Of these, 15 were excluded (7 not meeting the inclusion criteria and 8 declining participation), and 15 participants were enrolled in the study. Of the seven ineligible individuals, six did not meet the predefined criteria because of current sedative-hypnotic or psychiatric medication use and one because of a previously diagnosed sleep disorder. Among the eight who declined, six were unable to accommodate the EEG assessment (its duration and the required scalp preparation) and two were unwilling to use the headphone device required for BDAS. All enrolled participants completed the 14-day BDAS intervention and both pre- and post-intervention ISI and EEG assessments, with no withdrawals or loss to follow-up. No adverse events related to BDAS were reported.

Participant characteristics are summarized in Table 1. The mean age was 40.8 years (SD 9.7), and most participants were female (93.3%). Participants were primarily nurses (73.3%), followed by physicians (20.0%) and other healthcare staff (6.7%). Three participants (20.0%) performed shift work. The mean baseline ISI score was 10.0 (SD 5.55); five participants (33.3%) had ISI scores in the 0–7 range, six (40.0%) had mild insomnia (ISI 8–14), and four (26.7%) had moderate-to-severe insomnia (ISI 15–28). Thus, the sample represented healthcare professionals with a spectrum of subjective sleep disturbances, rather than a cohort restricted to chronic insomnia.

**Table 1:** Baseline characteristics of participants (*n* = 15).

Characteristic	Value
Age, years	40.8 ± 9.7
Sex, *n* (%)	
Female	14 (93.3%)
Male	1 (6.7%)
Occupation, *n* (%)	
Nurse	11 (73.3%)
Physician	3 (20.0%)
Other healthcare staff	1 (6.7%)
Shift work, *n* (%)	3 (20.0%)
Baseline ISI score	10.0 ± 5.55
Insomnia severity category, *n* (%)	
No clinically significant insomnia (ISI 0–7)	5 (33.3%)
Mild insomnia (ISI 8–14)	6 (40.0%)
Moderate-to-severe insomnia (ISI ≥ 15)	4 (26.7%)

Values are presented as mean ± standard deviation or number (percentage), as appropriate. ISI, Insomnia Severity Index.

### 3.2 Subjective insomnia severity

A total of 15 participants completed both baseline and post-intervention ISI assessments. The mean ISI score decreased from 10.00 ± 5.55 at baseline to 4.27 ± 2.69 after the 14-day intervention. The mean within-participant change was −5.73 points (95% CI −8.15 to −3.32), corresponding to a mean reduction of 5.73 points. This within-participant change was statistically significant in a paired-sample *t* test, *t*(14) = −5.09, *p* < 0.001, with a standardized within-participant effect size of Cohen’s dz. = 1.31 and Hedges’ gz = 1.24. A Wilcoxon signed-rank test was additionally performed as a non-parametric sensitivity analysis and yielded W = 1.00, *p* < 0.001. The rank-biserial effect size was 0.98.

**Figure 3:**
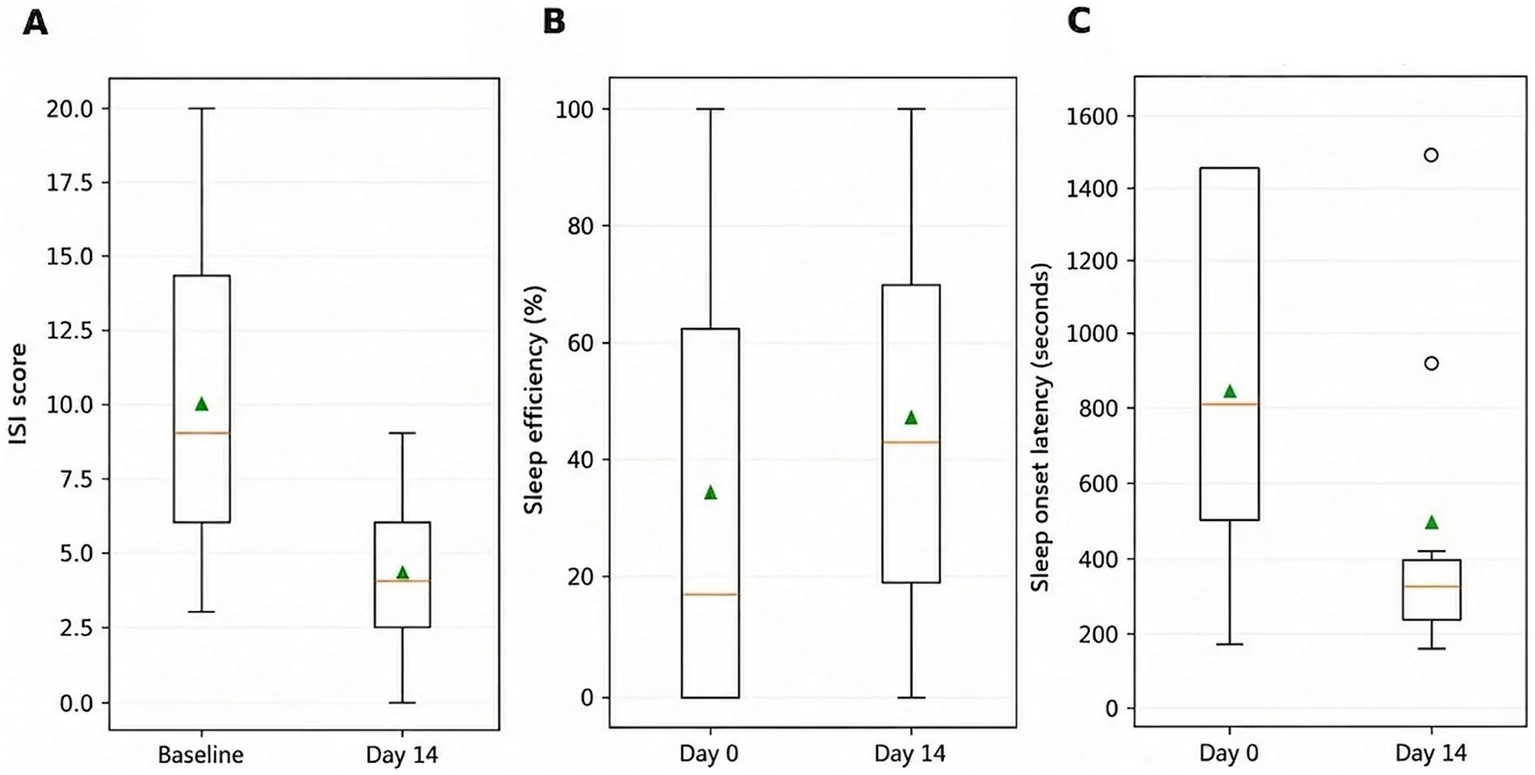
Changes in subjective insomnia severity and EEG-derived sleep parameters following the 14-day Brain Dynamic Audio Stimulation (BDAS) intervention. **(A)** Distribution of Insomnia Severity Index (ISI) scores at baseline and Day 14. **(B)** Distribution of EEG-derived sleep efficiency (SE%) during the 30-min recording period at baseline (Day 0) and Day 14. **(C)** Distribution of EEG-derived sleep onset latency (SOL) during the 30-min recording period at baseline (Day 0) and Day 14. In all panels, boxes represent the interquartile range (IQR), horizontal lines indicate medians, whiskers extend to the most extreme non-outlier values, circles represent outliers, and green triangles denote mean values. Following the 14-day BDAS intervention, participants demonstrated lower ISI scores, higher sleep efficiency, and shorter sleep onset latency.

The mean reduction approached the commonly cited 6-point threshold for clinically meaningful improvement on the ISI. Because this was a single-arm study without a control condition, the finding represents an uncontrolled within-participant change and does not establish a BDAS-specific effect. The distribution of ISI scores at baseline and Day 14 is shown in Figure 3A.

### 3.3 EEG-derived sleep outcomes

In the primary paired day-0 to day-14 analysis, 15 participants had paired short-duration EEG-derived outcomes. Both sleep efficiency and sleep onset latency showed favorable changes from baseline to day 14. Sleep efficiency during the 30-min EEG recording window increased from 34.76 ± 39.21% at day 0 to 46.83 ± 33.46% at day 14. The mean within-participant change was 12.06 percentage points (95% CI 0.34 to 23.79). This change was statistically significant in the paired-sample t test (*p* = 0.045) and remained consistent in the Wilcoxon signed-rank test (*p* = 0.034), with a moderate within-participant effect size (Cohen’s dz. = 0.57; Hedges’ gz = 0.54). Sleep onset latency decreased from 828.00 ± 499.79 s at day 0 to 480.00 ± 430.13 s at day 14. The mean within-participant change was −348.00 s (95% CI −565.05 to −130.95), corresponding to an average reduction of approximately 5.8 min. This change was statistically significant in the paired-sample t test (*p* = 0.004) and remained consistent in the Wilcoxon signed-rank test (*p* = 0.002), with a moderate-to-large within-participant effect size (Cohen’s dz. = 0.89; Hedges’ gz = 0.84). The distribution of sleep efficiency and sleep onset latency values is presented in Figures 3B,C.

No unintended consequences of the intervention were observed. No participants reported daytime sedation, cognitive impairment, or interference with work performance, and no technical problems with headphone use or audio delivery were reported.

## 4 Discussion

This single-group pre–post pilot feasibility study found reductions in insomnia symptom severity and favorable changes in short-duration EEG-derived sleep parameters after the two-week BDAS intervention among healthcare professionals with subjective sleep disturbances. Within this small sample, mean ISI scores decreased substantially over 14 days, and short-window EEG assessments during BDAS exposure captured sleep-stage transitions and an increase in 30-min sleep efficiency across participants. These findings indicate that BDAS is feasible and acceptable in a healthcare occupational setting and provide preliminary estimates of potential effect sizes for subjective and EEG-based sleep outcomes to inform future randomized controlled trials.

The mean within-participant ISI reduction was 5.73 points, approaching the commonly cited 6-point threshold for minimal clinically important difference. Because the study lacked a control group, this magnitude should be used primarily to inform future trial planning rather than compared directly with effect estimates from controlled trials. Established interventions such as cognitive behavioral therapy for insomnia remain first-line treatments in clinical guidelines (Morin and Benca, 2012; Riemann et al., 2023). Long-term hypnotic use raises concerns regarding dependence and residual daytime sedation; associations with other adverse outcomes are largely observational and may reflect residual confounding. In primary care populations, stronger hypnotic use has been associated with higher perceived stress and insomnia severity (De Las Cuevas and Segovia Díaz, 2025; Kalmbach et al., 2018; Kalmbach et al., 2018). Given the high prevalence of insomnia among healthcare professionals, BDAS could therefore be evaluated as a low-burden adjunct to existing sleep health strategies. Approximately one-third of participants (*n* = 5, 33.3%) had baseline ISI scores in the subclinical range (ISI 0–7) and did not meet conventional thresholds for clinically significant insomnia. Floor effects in these participants may have constrained the observable change and increased uncertainty, and the sample is not representative of populations with established insomnia disorder. The findings apply most directly to healthcare professionals reporting subjective sleep disturbances.

The short-duration physiological recordings provided objective, AASM-scored sleep-staging context that complemented the patient-reported ISI findings. Sleep-stage transitions occurred during the 30-min recording windows, and 30-min sleep efficiency increased from baseline to Day 14. Blinded scoring reduces observer-related scoring bias, but these EEG-derived outcomes do not independently confirm efficacy and remain subject to the limitations of the uncontrolled pre-post design. These observations also do not demonstrate cortical entrainment or a BDAS-specific neurophysiological mechanism because quantitative spectral analysis, stimulus-EEG phase-locking analysis, and a sham or non-modulated auditory control were not included. The findings should therefore be regarded as hypothesis-generating and require direct testing in future controlled studies.

These results should be interpreted within the broader context of auditory neuromodulation research. Systematic reviews of binaural-beat stimulation report heterogeneous effects on EEG activity and limited evidence for robust frequency-specific entrainment (Askarpour et al., 2024; Ingendoh et al., 2023). Rhythmic, closed-loop, and phase-locked auditory stimulation paradigms have used different approaches to influence sleep-related oscillations. Some closed-loop and phase-locked systems use ongoing EEG activity to determine stimulus timing, whereas earlier rhythmic paradigms delivered stimulation according to predefined temporal patterns (Ngo et al., 2013; Leminen et al., 2017; Papalambros et al., 2017; Besedovsky et al., 2017; Bressler et al., 2024). These approaches differ materially from the open-loop BDAS protocol. A recent randomized controlled trial of closed-loop acoustic neuromodulation in healthcare workers reported improvements in perceived stress, with insomnia and anxiety as secondary outcomes (Tegeler et al., 2025). These cross-study comparisons are indirect and descriptive because the populations, intervention components, study designs, and control conditions differ; they should not be interpreted as evidence of comparable efficacy.

BDAS was developed on the experimental hypothesis that the open-loop auditory stimulus might interact with sleep-related neural dynamics. The present study did not directly test this hypothesis and cannot establish cortical entrainment or a specific mechanism of action. Ho et al. (2025) is cited as prior exploratory background rather than definitive mechanistic evidence. Future mechanistic studies should include quantitative stimulus-synchronized EEG power and phase-locking analyses together with an appropriate sham or non-modulated auditory control.

The pilot findings support progression to a future randomized controlled trial, as all predefined progression criteria were met. All 15 enrolled participants completed the 14-day intervention and outcome assessments (100% retention; 0% discontinuation), and analyzable EEG data were successfully acquired for all paired recording sessions (100%), each exceeding the respective ≥80% thresholds. No serious adverse events were reported. The within-participant effect size for ISI reduction (Cohen’s dz. = 1.31) substantially exceeded the *a priori* threshold of d ≥ 0.5, and statistically significant improvements were observed in both EEG-derived sleep efficiency (dz = 0.57) and sleep onset latency (dz = 0.89), providing provisional estimates to inform planning of a future trial rather than evidence of efficacy. The single-center design and modest sample size nonetheless limit generalizability, and multicenter studies will be required to confirm feasibility across diverse healthcare environments.

Several limitations warrant consideration. The single-group pretest-posttest design without a control or sham condition precludes causal inference. The self-reported ISI outcome may be particularly susceptible to expectancy or placebo effects. The EEG-derived outcomes were based on physiological recordings scored according to AASM criteria by scorers blinded to assessment time point, which reduces self-report and observer-related scoring bias; however, objective measurement does not eliminate expectancy-related behavioral changes, familiarization with the recording environment, regression to the mean, or spontaneous symptom fluctuation. Exploratory baseline-severity subgroups were also very small, including only four participants with moderate-to-severe insomnia, and subgroup patterns cannot establish differential efficacy. The modest overall sample size limits generalizability and makes the effect-size estimates imprecise and provisional rather than definitive indicators of efficacy. The predominantly female sample may further limit generalizability. EEG assessments were restricted to a 30-min stimulation window in a hospital setting rather than full-night polysomnography or habitual sleep at home. Accordingly, 30-min sleep efficiency is not equivalent to conventional whole-night sleep efficiency and does not provide a complete assessment of sleep architecture. Retaining the fixed 30-min denominator when residual artifact-affected epochs were excluded from staging may conservatively underestimate this outcome. Daytime functioning, mood, occupational performance, and medication use were not systematically assessed, and the durability of the observed changes remains unknown.

Future research should employ randomized, sham-controlled, multicenter designs to distinguish BDAS-specific effects from placebo or expectancy influences and to clarify the specificity of BDAS-associated changes. The control condition could consist of non-modulated audio or pink noise matched for duration and delivery, and, where feasible, both participants and assessors should be blinded to reduce expectancy and outcome-assessment bias. To ensure at least mild insomnia at baseline and reduce the heterogeneity observed in this feasibility sample, future trials should enroll participants meeting a minimum insomnia threshold (for example, ISI ≥ 8). Potential moderators such as shift-work status and occupational role should also be evaluated, given that occupational schedules could influence treatment response. Objective assessments should be strengthened to include actigraphy, full-night polysomnography—in at least a subset of participants, to evaluate sleep architecture—and autonomic measures such as heart rate variability, together with extended follow-up to establish the durability of any benefit. Collectively, these findings support the feasibility of a larger randomized controlled trial to evaluate the efficacy and mechanisms of BDAS.

## 5 Conclusion

This pilot study observed reductions in insomnia severity and favorable changes in early sleep-related EEG parameters following the 14-day BDAS intervention among healthcare professionals with subjective sleep disturbances. While these findings are preliminary and exploratory, and cannot be attributed to BDAS specifically in the absence of a control condition, they support further evaluation of BDAS as a feasible, low-burden, non-pharmacological approach in high-stress occupational settings. Randomized controlled trials with larger samples and comprehensive sleep assessments are needed to confirm efficacy and clarify underlying mechanisms.

## Data Availability

The raw data supporting the conclusions of this article will be made available by the authors, without undue reservation.
